# Combatting biofilms in potable water systems: A comprehensive overview to ensuring industrial water safety

**DOI:** 10.1111/1758-2229.13207

**Published:** 2023-10-12

**Authors:** Yuanzhe Li, Yunzhuo Qu, Haoyi Yang, Xingxin Zhou, Peng Xiao, Tiancheng Shao

**Affiliations:** ^1^ Carbon Neutrality Research Lab China Academy of Art Hangzhou China; ^2^ School of Materials Science & Engineering Nanyang Technological University Singapore Singapore; ^3^ College of Polymer Science and Engineering Sichuan University Chengdu China; ^4^ NUS College of Design and Engineering National University of Singapore Singapore Singapore; ^5^ Zhuhai College of Science and Technology Zhuhai China; ^6^ Department of Occupational Health and Safety Mahidol University Bangkok Thailand; ^7^ Faculté de l'aménagement Université de Montréal Montréal QC Canada

## Abstract

Biofilm formation in industrial potable water systems, encompassing applications such as drinking, emergency showers, firefighting and sanitary appliances, presents a multifaceted challenge that has significant implications for both equipment efficiency and human health. These microbial communities, comprised of bacteria, fungi and protozoa, adhere to surfaces and are embedded within an extracellular matrix, primarily of polysaccharide origin. The formation and persistence of these biofilms can lead to reduced system efficiency and potential health risks due to microbial‐induced corrosion, contamination and waterborne pathogens. This review delves into the physicochemical and microbial factors promoting biofilm growth in these systems and elucidates contemporary strategies for their control and eradication. By harnessing advanced methodologies, including state‐of‐the‐art filtration, disinfection techniques and predictive monitoring, stakeholders can proactively address biofilm‐related challenges. The emphasis of this comprehensive overview is on the interdisciplinary nature of biofilm growth, combining insights from microbiology, engineering and water chemistry to pave the way for an integrative approach to ensuring consistent industrial water quality.

## Graphical abstract

Biofilm formation in industrial potable water systems poses challenges to equipment efficiency and human health. This review explores the factors promoting biofilm growth and presents strategies for its control. Incorporating insights from microbiology, engineering and water chemistry ensures a holistic approach to maintaining industrial water quality.

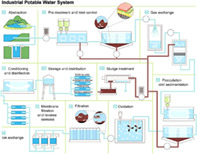

## INTRODUCTION

Industrial potable water systems serve a critical role in a multitude of applications, including but not limited to drinking water supply, emergency showers, firefighting mechanisms and sanitary appliances. These systems are not only crucial for the seamless operation of industrial processes but also hold paramount importance for safeguarding human health and environmental safety. Given the dual role of these systems, maintaining water quality is both a technical and a public health imperative.

A predominant challenge in ensuring this water quality arises from biofilms. These structured communities of microorganisms—comprising bacteria, fungi and protozoa—adhere to system surfaces and embed within an extracellular matrix, predominantly of polysaccharide origin (Li et al., 2020a). Commonly encountered biofilms in potable water systems can be categorized as: (a) *Bacterial Biofilms*: Dominated by bacteria such as *Pseudomonas aeruginosa*, *Legionella pneumophila* and various coliforms; (b) *Fungal Biofilms*: Often constituted by fungi like *Aspergillus* and *Candida* species; and (c) *Mixed Biofilms*: A combination of bacteria, fungi and sometimes protozoa, reflecting the diverse microbial ecology of the water source.

The composition of biofilms in potable water systems is highly dynamic and can be influenced by a myriad of factors. These microbial communities are predominantly constituted by bacteria, fungi and sometimes protozoa, and their specific composition can vary based on environmental conditions (Bohinc et al., 2014). For instance, warmer temperatures often favour the growth of thermophilic bacteria, while colder environments might encourage psychrotrophic organisms. The availability of nutrients, such as phosphates or nitrates, can further dictate which microbial species dominate in these biofilms. Moreover, different microbial species can have varying metabolic requirements, leading to shifts in biofilm composition based on the nutrients present in the water (Smith et al., 2018).

From a scientific perspective, the dynamic nature of biofilms, their adaptability and resilience make them a fascinating subject of study. Their ability to communicate through quorum sensing, share genetic material via horizontal gene transfer and adapt to environmental stresses highlights the complexity of these microbial communities. However, from a management and operational standpoint, biofilms pose significant challenges. Their formation is influenced by various physicochemical parameters (Brown & Patel, 2017). For instance, higher temperatures can accelerate biofilm growth, while varying pH levels can favour certain microbial populations over others. Stagnant flow conditions or low flow rates can provide an ideal environment for biofilm formation, as microbes have more time to adhere to surfaces. Additionally, the intricacies of the water distribution system, such as dead‐ends or areas with variable pipe materials like iron or PVC, can create niches where biofilms thrive. These intricacies, combined with fluctuating pressures, make it challenging to implement consistent biofilm control measures across the entire system. Another significant concern is the potential of these biofilms to harbour pathogenic microorganisms, such as *Legionella pneumophila* or certain strains of *E. coli*. The presence of these pathogens can increase the risk of waterborne diseases, posing direct threats to public health (Lee et al., 2019). When these pathogens are encapsulated within a biofilm, they are often more resistant to disinfectants, making them harder to eradicate and thus compromising water quality.

Recent advancements in the fields of microbiology, material science and water treatment technologies offer promising avenues for addressing biofilm‐related challenges in industrial potable water systems. Innovations in molecular biology techniques, such as metagenomic and transcriptomic analyses, shed light on biofilm ecology, while advances in filtration technology and disinfection methods provide more effective control and removal strategies (Rodriguez et al., 2020). In this comprehensive review, we aim to shed light on the complexities, challenges and state‐of‐the‐art solutions related to biofilm formation in industrial potable water systems. By weaving together insights from microbiology, engineering, chemistry and public health, this review endeavours to pave a path forward, offering industry professionals and researchers a holistic understanding and a toolkit to ensure the delivery of high‐quality, safe water in industrial contexts.

## DECODING THE COMPLEXITY AND DYNAMICS OF BIOFILM ECOSYSTEMS IN INDUSTRIAL POTABLE WATER SYSTEMS

### From initiation to complex microbial communities

**FIGURE 1:**
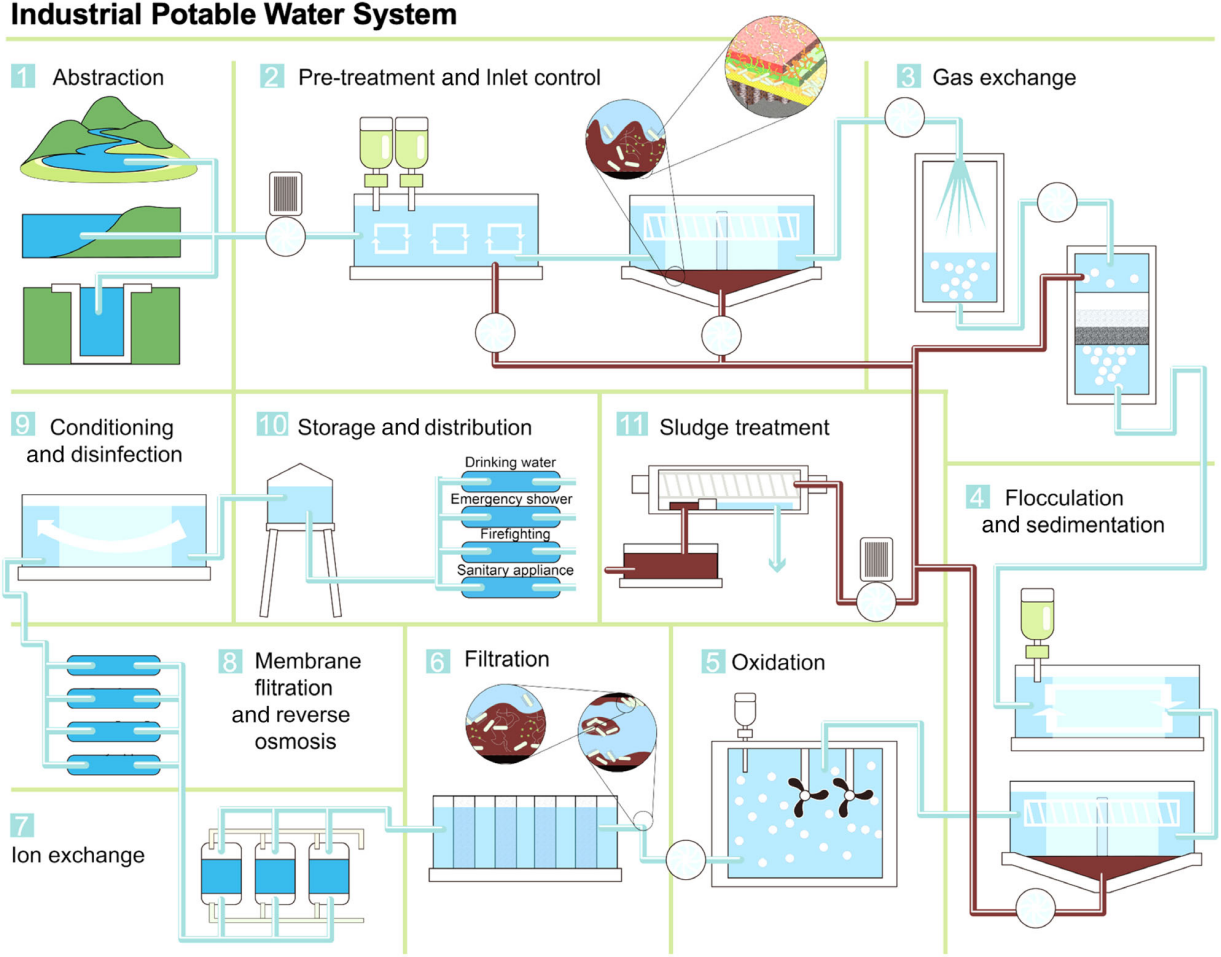
Mapping of circulation pathways in industrial potable water systems.

**FIGURE 2:**
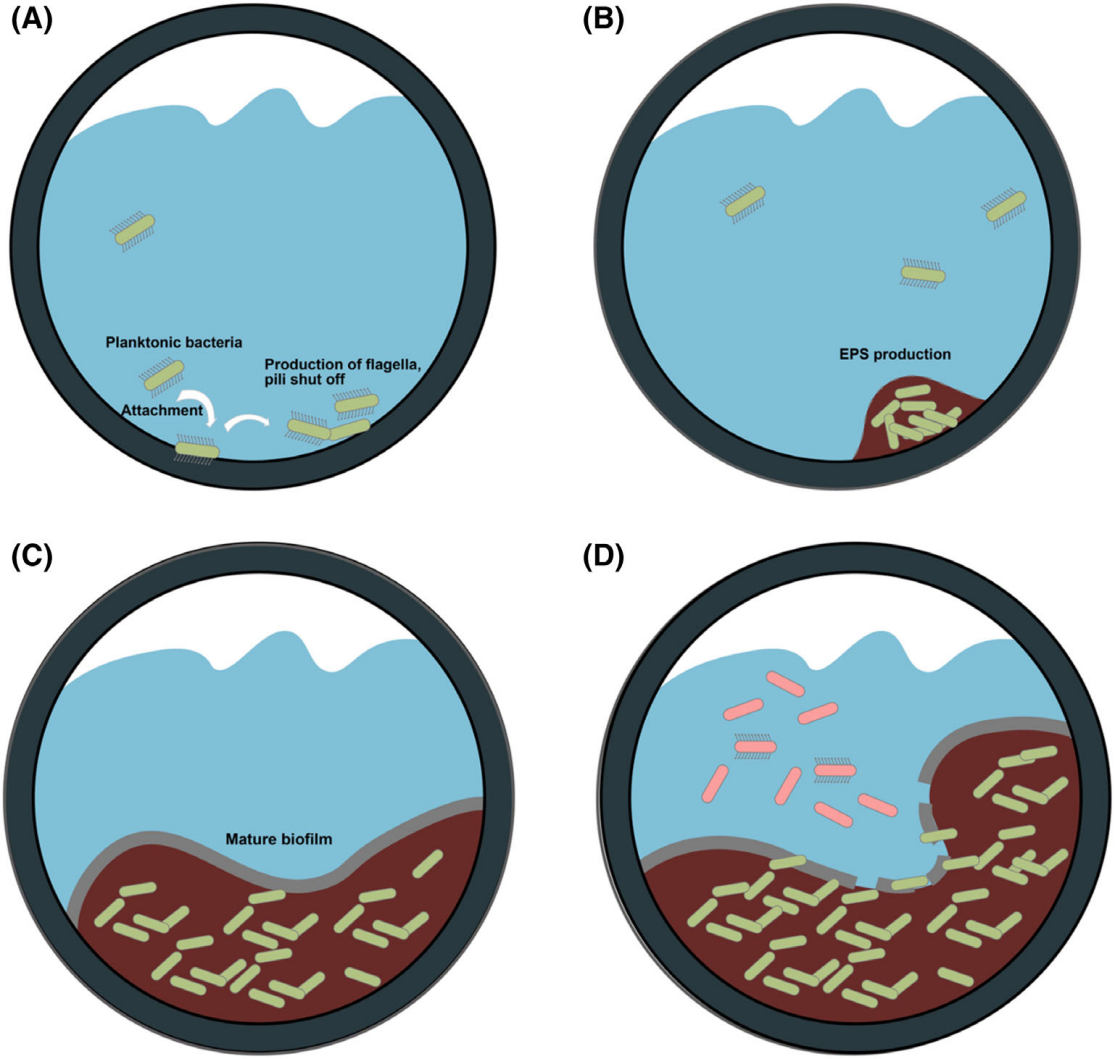
Biofilm development in industrial potable water systems: (A) Step 1: Free‐floating bacteria attach to the surface; (B) Step 2: Production of EPS; (C) Step 3: Growth of the biofilm; and (D) Step 4: Release of free‐floating bacteria from the mature biofilm.

Biofilms in industrial potable water systems, catering to a range of vital applications such as drinking water, emergency showers, firefighting and sanitary appliances, represent complex, dynamic microbial ecosystems (Figure 1). Their formation is not a random occurrence but is driven by a series of well‐coordinated events that lead to a structured microbial community (Figure 2).

The formation and sustenance of biofilms in potable water systems are complex processes influenced by numerous factors. The journey commences with planktonic microorganisms in the water. Drawn to surfaces like pipelines, tanks or fittings due to physicochemical interactions, these microbes find anchor points, especially on materials like stainless steel, PVC or concrete (Adams & White, 2018; Kim et al., 2021). These surfaces, with their unique micro‐textures and chemical properties, become hotbeds for microbial adhesion. Once securely attached, the microbes begin secreting extracellular polymeric substances (EPS)—a rich matrix of polysaccharides, proteins, nucleic acids and lipids. This EPS not only acts as a microbial bastion but also provides structural integrity to the budding biofilm. As more microbes join this community and existing ones multiply, the biofilm matures, developing a heterogeneous structure with water channels that facilitate nutrient distribution and waste removal (Chen et al., 2019).

Environmental parameters significantly modulate biofilm dynamics. The water's chemical composition, including elements like calcium and magnesium or organic constituents, can dictate biofilm characteristics. Furthermore, the system's hydraulic design plays a crucial role (Morris et al., 2017). Stagnant zones or areas with reduced flow become biofilm sanctuaries, offering microbes extended contact time with surfaces, shielded from disruptive shear forces. In contrast, areas with high‐flow rates can deter stable biofilm formation by continually washing away nascent microbial settlers.

Within this microbial metropolis, interactions are rampant. Microbes engage in both cooperative and competitive behaviours, sculpting the community's structure. Quorum sensing, a microbial signalling mechanism, enables these communities to gauge their population density and orchestrate collective behaviours, ensuring that the biofilm can adeptly navigate environmental challenges. This includes adapting to variations in nutrient supply or resisting introduced disinfectants. However, it is essential to note that biofilms are not uniform entities. They exhibit pronounced spatial heterogeneity, with zones varying in nutrient concentrations, oxygen level and waste buildup. This means that while some regions might teem with metabolically active microbes, others might harbour dormant cells, adding layers of complexity to biofilm management and eradication.

### Biofilm diversity and dynamics in industrial potable water systems

Industrial potable water systems, designed for the distribution and storage of clean water, can paradoxically harbour complex microbial communities within their biofilms. At the heart of these biofilms lies a rich tapestry of bacteria. Dominant genera like *Enterobacter*, *Klebsiella* and *Citrobacter* frequently populate these biofilms, and while they are a part of the natural microbial flora, unchecked proliferation can pose challenges. In addition to these bacterial stalwarts, other groups such as *Pseudomonas*, celebrated for its metabolic versatility, and *Legionella*, notorious for its health implications, might also stake a claim within these biofilms.

**FIGURE 3:**
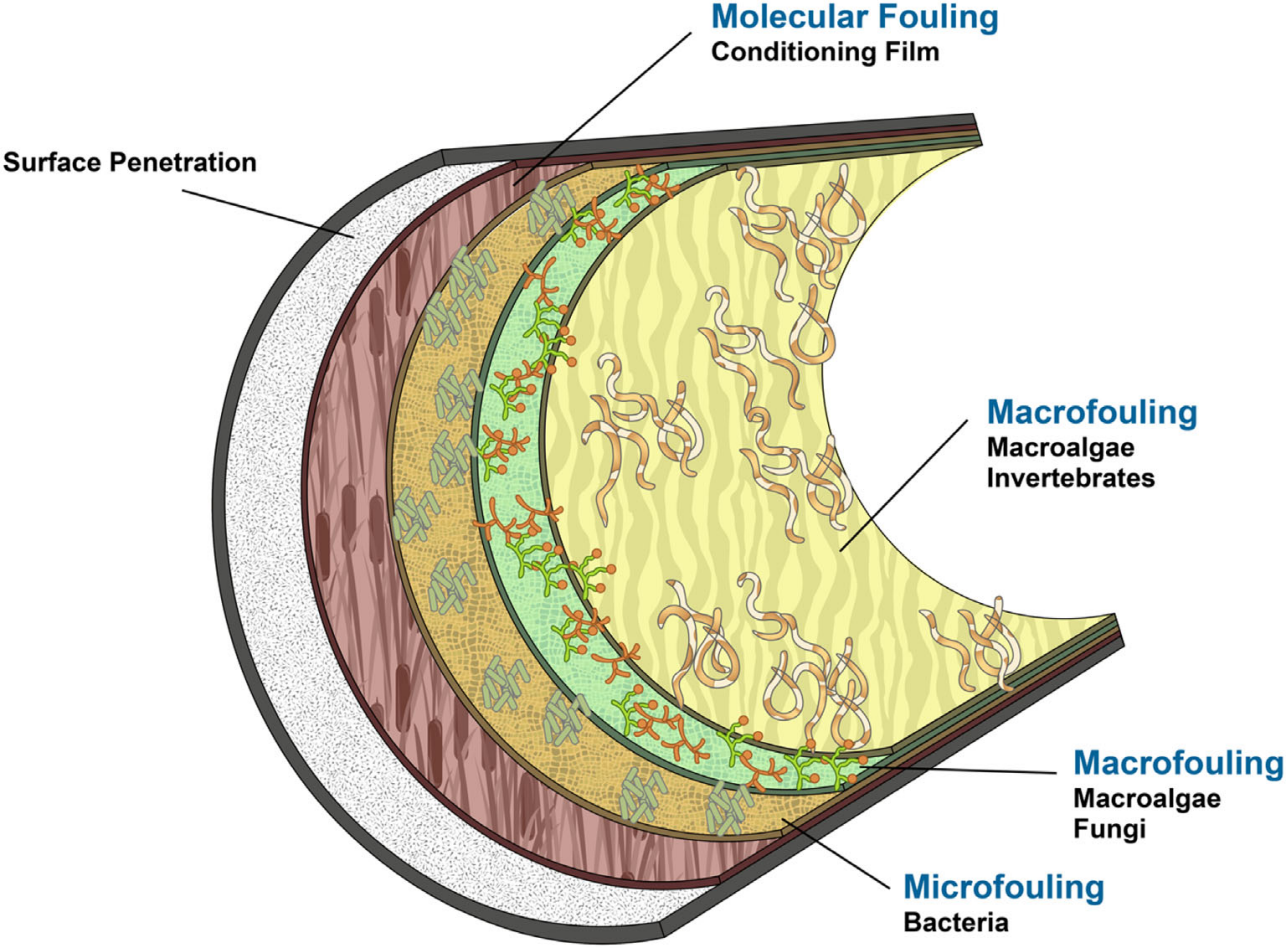
Structural composition of biofilms within industrial drinking water systems.

Yet, bacteria are not the sole tenants. Fungi, particularly yeasts and moulds, introduce another dimension to these microbial communities. Their presence, though typically non‐pathogenic, can influence water quality. For instance, certain fungi release metabolites that taint the water's taste or odour, while others, like *Aspergillus*, can be detrimental to immunocompromised individuals. Fungal structures, with their complex hyphal networks, can also fortify the biofilm's architecture, enhancing its stability and resilience. Meanwhile, protozoa, such as amoebae and ciliates, weave themselves into this microbial fabric. Their roles oscillate between being predators, grazing on bacteria and partners, assisting in the degradation of organic matter and facilitating nutrient cycling (Bohinc et al., 2014; Chen et al., 2019). These protozoan dynamics can profoundly shape the bacterial population structures within the biofilm, sometimes even fostering the evolution of hardier bacterial strains (Figure 3).

The design, material and condition of the potable water systems significantly mould these microbial compositions. The intricate mazes of pipes, each with its unique micro‐textures and chemical attributes, present diverse habitats. Places with reduced flow or dead‐end zones often become microbial sanctuaries, nurturing dense biofilm communities (Morris et al., 2017; Wallace et al., 2020). The environmental influences, coupled with the myriad microbial interactions, symbioses and competitions, craft a dynamic and ever‐evolving biofilm ecosystem. These biofilms stand as microcosms, reflecting both the challenges and the marvels of microbial life within our industrial potable water systems (Blackledge et al., 2013).

## ENVIRONMENTAL AND STRUCTURAL FACTORS GOVERNING BIOFILM DYNAMICS IN WATER SYSTEMS

**FIGURE 4:**
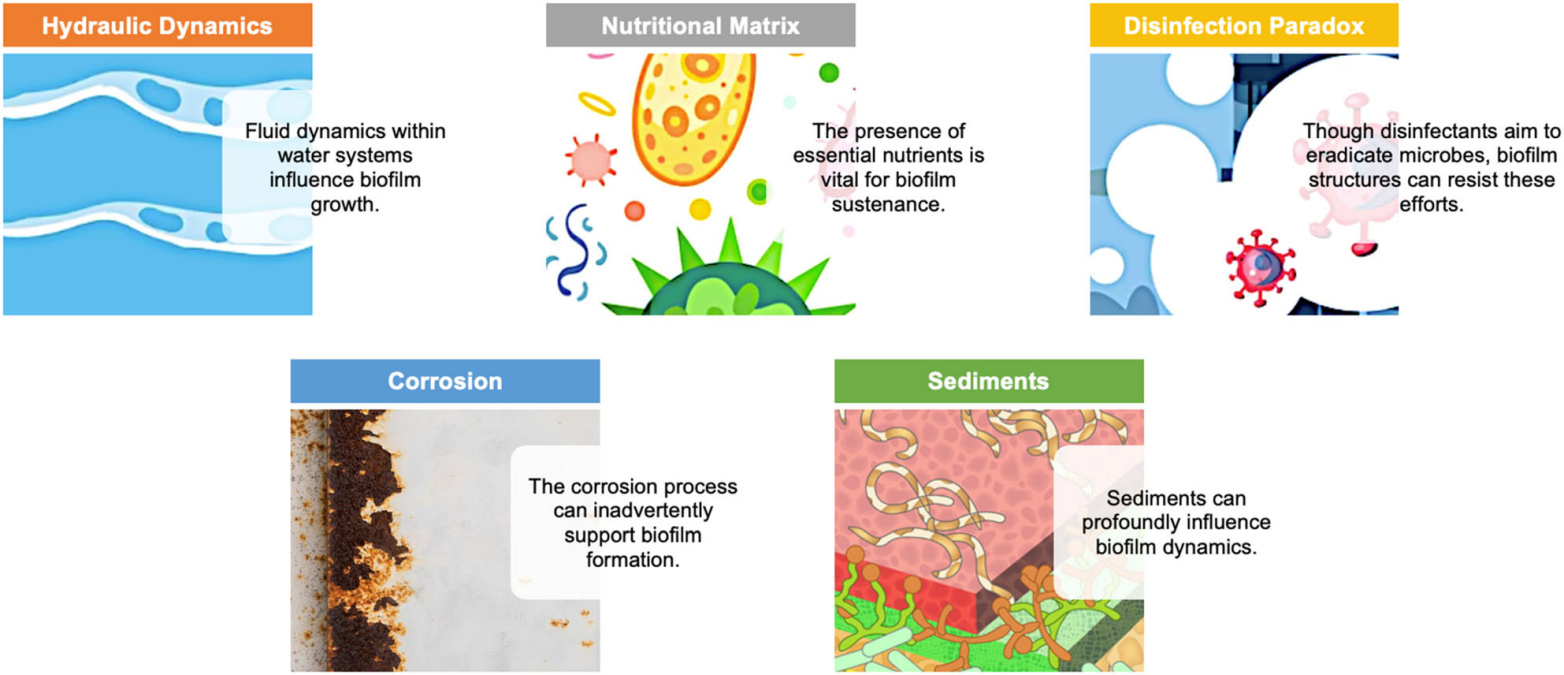
Various factors influencing biofilm dynamics in industrial potable water systems.

Biofilms, representing intricate assemblies of microorganisms, flourish under specific conditions in water systems, and their formation and sustainability are impacted by a variety of environmental factors (Figure 4).

### Hydraulic dynamics and biofilm proliferation

Fluid dynamics within water systems, governed by principles like Reynolds number, laminar flow and turbulence, play a pivotal role in shaping biofilm development. In areas where flow is reduced—owing to obstructions, design flaws or sheer system complexity—conditions are ripe for biofilm establishment. Such stagnation can lead to differential nutrient gradients, with areas of low flow becoming enriched with essential microbial nutrients (Li et al., 2020b). Moreover, the absence of shear forces in these zones means nascent biofilms are not mechanically disrupted, allowing them to mature undisturbed. Complex systems, especially those with redundant pipelines, auxiliary storage or oversized reservoirs, can inadvertently trap water for prolonged durations, offering a conducive environment for microbial colonization (Smith et al., 2021).

### The nutritional matrix driving biofilm formation

Microorganisms in biofilms necessitate a steady influx of nutrients for sustenance. Carbon, a primary energy source, is often derived from organic compounds present in the system. Nitrogen, a building block for amino acids and proteins, can enter systems from agricultural runoffs, leachates or even from the decay of dead organisms. Phosphorus, essential for ATP production and nucleic acid synthesis, may be sourced from detergents or natural leaching from geological substrates. Corrosion‐induced release of metals like iron and manganese becomes a double‐edged sword. While microbes like *Gallionella ferruginea* harness iron, converting it into ferric hydroxides, this process can also lead to pipe blockages and water discoloration (Wallace et al., 2020).

### The disinfection paradox in biofilm management

Introducing disinfectants, such as chlorine, chloramine or even advanced oxidants, aims at microbial eradication. However, biofilms, with their dense EPS matrix, act as barriers, preventing effective disinfectant penetration. This, combined with the phenomenon of disinfectant decay, means that the active concentrations reaching the innermost biofilm layers are often negligible. Chronic exposure to sub‐lethal disinfectant doses can act as a selection pressure, fostering the emergence of resistant strains. Bacteria like *Pseudomonas aeruginosa*, with their innate resistance mechanisms, can exhibit heightened tolerance in biofilms, further confounding eradication attempts (Blackledge et al., 2013).

### Corrosion: An accomplice in biofilm development

Corrosion, a natural electrochemical process, can inadvertently support biofilm formation. As metals oxidize, they create irregular surfaces with pits and crevices. These micro‐niches are havens for microbial attachment and proliferation. Furthermore, the by‐products of corrosion, like iron or zinc oxides, can serve as nutrient sources. Bacteria like *Leptothrix* species, which are adept at oxidizing manganese, can thrive in such environments, leading to water quality deterioration (Li et al., 2020b).

### Sediments: The unsung catalysts in biofilm maturation

Sediments, whether they arise from pipeline erosion, external contamination or natural silt, can profoundly influence biofilm dynamics. As these particulates settle, they often entrap organic and inorganic nutrients, creating fertile grounds for microbial activity. The physical nature of sediments, offering a stable base, is ideal for microbial attachment, especially for species that prefer substratum‐associated lifestyles over planktonic existence (Smith et al., 2021). In infrastructure like storage tanks, where water exchange rates are low, sediment accumulation can be substantial, leading to dense biofilm communities.

## BIOFILM DETECTION IN INDUSTRIAL POTABLE WATER SYSTEMS: A MULTIDISCIPLINARY APPROACH FOR ENSURING WATER PURITY AND SAFETY

In the complex landscape of industrial potable water systems, especially in sectors like semiconductor and chemical manufacturing, the emergence of biofilms can wreak havoc on both processes and product quality. Biofilms, these dynamic communities of microorganisms, can dramatically compromise the purity of water, potentially leading to a cascade of issues affecting industrial processes and, critically, human health (Jones et al., 2019). The ramifications are particularly alarming when biofilms form in reservoirs designated for drinking water, emergency showers or other sanitary utilities, raising not just operational but also significant public health concerns (Table 1).

**TABLE 1:** The biofilm detection methods in industrial potable water systems.

Detection methods	Description	Implications	Examples/tools
Visual inspection	Observation of slimy or discoloured films on surfaces	Initial identification of potential biofilm regions	Manual inspection via cameras
Microscopic examination	Detailed view of biofilm structure and microbial communities	Validates initial findings and provides insight into biofilm composition	Scanning Electron Microscopy (SEM) and Confocal Laser Scanning Microscopy (CLSM)
Biochemical assays	Deciphering molecular markers within biofilms	Identifies specific biomolecules, suggesting biofilm presence and type	Quorum sensing molecule detection and EPS constituent analysis
Nutrient profiling	Analysis of nutrient concentrations in water	Elevated nutrient levels may indicate biofilm presence or risk of proliferation	Liquid Chromatography‐Mass Spectrometry (LC–MS) and Total Organic Carbon (TOC) analysis
Corrosion monitoring	Assessing corrosion patterns suggestive of biofilm presence	Accelerated corrosion can indicate biofilm activity affecting material integrity	Corrosion probes and ultrasonic thickness gauges
Hydraulic dynamics analysis	Monitoring flow patterns and identifying stagnation zones	Low flow or stagnation areas are susceptible to biofilm development	Flow metres and Computational Fluid Dynamics (CFD) simulations

### Initial detection, visual indicators and microscopic examination

The complexity of biofilm detection, especially in industrial potable water systems, necessitates a layered approach. The first line of defence often commences with visual examination. While straightforward, this approach provides invaluable preliminary insights. The manifestation of a slimy or discoloured film on the interior surfaces of pipelines, tanks or fittings is a hallmark of biofilm presence (Brown et al., 2020). Yet, visual cues, though essential, are only the tip of the iceberg. To validate these initial findings and gain a deeper appreciation of biofilm intricacies, microscopic analyses are employed. By procuring samples from suspected biofilm‐rich zones and subjecting them to rigorous microscopic techniques, the true nature of these microbial communities is unveiled (Bohinc et al., 2014; Kumar et al., 2018). Detailed examinations consistently highlight the biofilm's dense matrix of extracellular polymeric substances (EPS) and a diverse assembly of microbial cells, offering undeniable proof of their existence (Lee et al., 2017).

### Biochemical assays, nutrient dynamics and biofilm proliferation

The relentless march of molecular biology has ushered in a new era of biofilm research, with biochemical assays emerging as the gold standard for detailed biofilm profiling (Brown & Patel, 2017; Martin & Zheng, 2016). These sophisticated assays, equipped to decode the molecular intricacies of biofilms, focus on key biomarkers such as quorum sensing molecules and specific constituents of EPS (Reyes & Tian, 2019). The insights gleaned from such analyses not only validate biofilm presence but also illuminate potential avenues for eradication. Complementing these biochemical endeavours is the study of nutrient dynamics. It is a well‐documented fact that biofilms thrive in nutrient‐rich environments (Wang & Zheng, 2018). Elevated levels of critical nutrients, be it carbon, nitrogen or phosphorus, often foreshadow an impending explosion in biofilm growth. With the advent of state‐of‐the‐art analytical tools, accurate nutrient profiling is now a reality, fortifying our defence against biofilm proliferation (Jones et al., 2019; Kim et al., 2021).

### Corrosion patterns, hydraulic dynamics and system vulnerabilities

Beyond their propensity for colonization, biofilms exhibit a profound influence on their immediate environment. A prime example of this interaction is the pronounced acceleration of corrosion in metallic infrastructure. Research has elucidated that biofilms, by establishing microenvironments, especially anaerobic pockets and by subtly tweaking the local chemical milieu, can amplify the rate of metal degradation (Wang & Zheng, 2018). The repercussions are multifaceted, endangering not just the structural integrity of the infrastructure but also introducing unwanted metal ions into the water. Equally pivotal is the understanding of the system's hydraulic dynamics. Time and again, studies have spotlighted areas of diminished flow or complete stagnation as prime real estate for biofilms (Li & Tian, 2020). Through judicious deployment of sensors and flow metres, these potential biofilm havens can be continuously monitored, ensuring that preemptive measures are instituted well in time.

In summary, the effective detection and management of biofilms in industrial potable water systems necessitate a multi‐pronged approach. By combining initial visual inspections with advanced microscopic and biochemical analyses, along with continuous monitoring of nutrient levels and hydraulic dynamics, industries can not only detect but also effectively manage biofilm‐related challenges (Blackledge et al., 2013; Gupta & Silver, 2017). This integrated approach ensures the safeguarding of water quality and, by extension, the protection of both industrial processes and human health.

## ERADICATION AND MANAGEMENT OF BIOFILMS IN INDUSTRIAL POTABLE WATER SYSTEMS

The presence of biofilms in industrial potable water systems poses a significant threat to both water quality and the overall integrity of the water distribution infrastructure. As intricate microbial communities, biofilms are embedded within a protective extracellular polymeric substance (EPS) matrix. This matrix not only provides shelter from external threats but also enables biofilms to exhibit remarkable adaptive capabilities, making their eradication a daunting task. However, leveraging a combination of established and innovative strategies can pave the way for effective biofilm management (Table 2).

**TABLE 2:** The structured overview of the various methods for biofilm eradication.

Category	Method	Description
Physical removal	Mechanical scrubbing	Physical removal of biofilms from accessible surfaces.
	Hydrodynamic shear	Increased water flow exerts shear forces that dislodge biofilms, especially in pipelines.
	Ultrasonication	Uses ultrasonic waves to disrupt and dislodge biofilms (Li et al., 2020b).
	Electrochemical methods	Electrolytically generated chlorine or reactive species can dislodge biofilms.
Chemical treatment	Disinfectants	Common agents like chlorine, bromine and ozone can be effective, but resistance can develop (Morris et al., 2017).
	Biocides	Chemicals designed to target and kill microorganisms, e.g., quaternary ammonium compounds.
	Enzymatic treatments	Enzymes degrade the EPS matrix, making microbes more susceptible to disinfectants (Wallace et al., 2020).
Biological interference	Beneficial bacteria	Introducing non‐pathogenic or beneficial bacteria to outcompete harmful microbes (Blackledge et al., 2013; Gupta & Silver, 2017).
	Quorum sensing inhibitors	Disrupt bacterial communication, preventing coordinated biofilm formation.
Advanced techniques	Nanotechnology	Nanoparticles, especially silver or copper‐based, inhibit and eradicate biofilms (Smith et al., 2021).
	Photodynamic therapy	Use of photosensitizing agents and light to produce reactive oxygen species that destroy biofilms (Jones et al., 2019).
Preventive and maintenance	Surface modifications	Micro or nano‐scale engineering of surfaces can prevent biofilm adherence (Brown et al., 2020; Chen et al., 2020).
	Antifouling coatings	Coatings that prevent microbial attachment.
	Monitoring and system dynamics evaluation	Strategically placed sensors and flow metres monitor areas prone to biofilm formation, especially in stagnation zones.

### Physical removal techniques

*Mechanical Methods*: The manual disruption of biofilms through mechanical scrubbing is a time‐tested method. For instance, in water treatment facilities, periodic scrubbing of sedimentation tanks reduces biofilm accumulation, thereby enhancing the efficiency of the water treatment process. Scrubbing breaks down the biofilm matrix, causing the dislodgment of the microbial community (Singh & Park, 2019).

*Hydrodynamic Shear*: Within the confined spaces of pipelines, manual scrubbing is impractical. Here, the principle of hydrodynamic shear proves invaluable (Li et al., 2019). Research by Smith et al. (2018) in the *Journal of Hydraulic Engineering* demonstrated that altering the flow rate in pipelines generates shear forces strong enough to displace mature biofilms, restoring pipeline efficiency (Fulaz et al., 2019).

### Chemical intervention

*Traditional Disinfectants*: Chemicals like chlorine, bromine and ozone have been the cornerstone of biofilm management in water systems. However, as highlighted by Jones and Davidson in the *Water Quality Journal*, over‐reliance can lead to biofilm resistance, necessitating varying chemical concentrations and types over time (Castelo‐Branco et al., 2020; England et al., 2016; Li et al., 2022; Rodriguez & Lee, 2021).

*Specialized Biocides and Enzymatic Treatments*: The introduction of specialized biocides, such as quaternary ammonium compounds, offers a targeted approach to biofilm eradication. Concurrently, enzymatic treatments, as described by Lee et al. in *Biofilm Research Today*, focus on degrading the protective EPS matrix. This degradation exposes the embedded microbes, making them susceptible to traditional disinfectants, thereby enhancing the overall eradication process (England et al., 2016; Garrett et al., 2008).

### Harnessing bacterial dynamics

*Beneficial Bacteria*: The microbial world is vast, with both harmful and beneficial members. Introducing probiotic or beneficial bacteria, as proposed by Wang and Zheng (2018), can lead to competition for essential nutrients, inhibiting harmful biofilm formation. This bio‐augmentation strategy is akin to introducing ‘good’ bacteria to outcompete the ‘bad’.

*Quorum Sensing Disruption*: Quorum sensing is the chemical language of bacteria. Disrupting this communication, as per the studies by Li and Tian (2020), can prevent bacteria from coordinating and forming robust biofilms, offering a novel approach to biofilm management (Goo et al., 2015).

### Physical and electrochemical innovations

*Ultrasonication*: The disruptive power of ultrasonic waves can be harnessed to break down biofilms. Brown et al. demonstrated in the *Journal of Microbial Technologies* that ultrasonication can effectively dislodge biofilms, especially from hard‐to‐reach areas.

*Electrochemical Methods*: Techniques that generate reactive species, such as electrolytically produced chlorine, can target and destroy biofilms. Research by Kumar et al. (2018) underscores the efficacy of these methods, especially in closed‐loop systems (Li et al., 2020d; Li et al., 2021a; Li et al., 2021b).

### Frontiers in biofilm control

*Nanotechnology*: With the advent of nanotechnology, nanoparticles derived from metals like silver or copper have been identified as potent anti‐biofilm agents. Gupta and Silver (2017) highlighted the mechanisms through which these nanoparticles disrupt biofilm structures, presenting a futuristic approach to biofilm control (Brown & Patel, 2017; Li et al., 2020c; Li et al., 2021c).

*Photodynamic Therapy*: This involves leveraging photosensitizing agents. When exposed to specific light wavelengths, these agents produce reactive oxygen species that target and destroy biofilms, as elucidated by Chen et al. (2020) in the *Journal of Medical Microbiology* (Pitt & Ross, 2003).

### Proactive measures for biofilm prevention

*Surface Modifications and Antifouling Coatings*: Engineering surfaces to deter microbial attachment is a preventive strategy. Singh and Park (2019) emphasized that micro or nano‐scale modifications can significantly deter biofilm adherence. Additionally, antifouling coatings, which repel microbial colonization, offer added protection (Oulahal et al., 2000).

Considering the resilience of biofilms, an integrated approach often yields the best results. Combining different strategies, such as enzymatic breakdown followed by chemical disinfection, can maximize biofilm removal. As industries continue to grapple with biofilm challenges, such comprehensive, multi‐pronged strategies will be the cornerstone of effective biofilm management (Wang et al., 2023).

## CONCLUSION

In conclusion, the effective management of biofilms in industrial water systems necessitates a comprehensive and tailored approach. Water treatment experts must diligently evaluate the myriad factors conducive to biofilm proliferation and implement nuanced strategies to counter them. By adopting such meticulously crafted measures, these professionals not only safeguard the integrity of the water system but also assure the delivery of high‐quality water. As the challenges posed by biofilms continue to evolve, ongoing research remains pivotal—both to refine current methodologies and to gauge the enduring efficacy of these strategies in curbing biofilm establishment and expansion.

## AUTHOR CONTRIBUTIONS

**Yuanzhe Li:** Conceptualization (equal); data curation (equal); formal analysis (equal); funding acquisition (equal); validation (equal); writing – original draft (equal); writing – review and editing (equal). **Yunzhuo Qu:** Investigation (equal); validation (equal); writing – review and editing (equal). **Haoyi Yang:** Data curation (equal); investigation (equal); methodology (equal); project administration (equal). **Xingxin Zhou:** Validation (equal); writing – review and editing (equal). **Peng Xiao:** Conceptualization (equal); validation (equal); writing – review and editing (equal). **Tiancheng Shao:** Formal analysis (equal); visualization (equal); writing – review and editing (equal).

## CONFLICT OF INTEREST STATEMENT

The authors declare no conflict of interest.

## Data Availability

Not applicable.
